# WHO FCTC: How we got here and where we are going

**DOI:** 10.18332/tid/203569

**Published:** 2025-04-15

**Authors:** Laurent Huber, Megan Arendt-Manning

**Affiliations:** 1 Action on Smoking and Health (ASH), Washington, United States

**Keywords:** WHO FCTC, Framework Convention on Tobacco Control, Framework Convention Alliance for Tobacco Control, tobacco control, civil society

When I began working at Action on Smoking and Health (ASH) in 2000, I was given a very broad mandate: do everything you can to support this tobacco treaty. The ASH Board had the foresight to recognize and understand the global significance of what would become the World Health Organization Framework Convention on Tobacco Control, the FCTC, and decided to fully stand behind the development of the first global health treaty.

At that time, tobacco was advertised everywhere, no country had comprehensive smoke-free policies, not a single country had pictorial health warnings, and plain packaging was barely a consideration. Many thought the concept of smoke-free bars and restaurants was going too far.

The FCTC was a game-changer in global health: it provided a clear path for countries on what to do to reduce the prevalence of tobacco use.

ASH strengthened the participation of civil society in the tobacco treaty process by supporting the Framework Convention Alliance (FCA)^1^, now the Global Alliance for Tobacco Control (GATC)^2^, an alliance of non-governmental organizations that was a powerful voice and had a tremendous impact on the FCTC process. ASH served as the secretariat of the FCA for seventeen years, and I had the privilege to serve as its first director^3^.

Through the years, the FCA was able to form a coalition of more than 500 public health, human rights, consumer rights, women’s and children’s rights organizations and environmental activists from over 100 countries, rallying to support the development of the strongest, evidence-based, and most effective FCTC^3^.

*‘I had the privilege to work closely with the FCA through the development of the FCTC and to witness firsthand the expertise they bring to the process of negotiating and adopting complex policy. The importance of having non-government and government agencies work together cannot be underestimated, and FCA understands very well how to influence governments to create the best possible policies’,* said Tábare Vázquez, President of Uruguay (2005–2010)^4^.

Working with a coalition of supportive countries, including India, Thailand, Canada, New Zealand, the island nations of the Pacific and Caribbean, and the entire continent of Africa, the FCA was able to thwart the desires of tobacco producing governments that sought a weak and non-binding treaty during the FCTC negotiations. In just a few years, from the beginning of the treaty negotiations to its adoption in 2003, the FCA grew tremendously in size and influence and had a very significant impact not only on the final text of the FCTC but also its rapid entry into force and later development of Guidelines and the Illicit Trade Protocol^3^.

FCA members participated in all negotiating sessions of the FCTC, working collaboratively with governments, the WHO, and other UN entities. FCA provided educational materials, hosted delegates’ briefings, wrote a daily newsletter during the negotiations, provided tobacco control expertise, and offered in-country and regional strategic support. The FCA and its members helped shape much of the public climate that provided momentum for international regulation of the tobacco industry and then helped achieve consensus on what measures needed to be taken to reduce tobacco use^3^.

Without the scientific, educational, media and organizing expertise of hundreds of public health, tobacco control, consumer, human rights, and other organizations worldwide, a strong FCTC Convention may not have been achieved^3^. Even a member of the US delegation to the FCTC Negotiations, Gregory Jacob, who did not agree with many of the FCA’s positions, recognized the effectiveness of the FCA in an article^5^ published in the Chicago Journal of International Law where he wrote:

*‘The NGOs in Geneva were well organized and outspoken. In fact, Action on Smoking and Health (ASH), Campaign for Tobacco Free Kids, and an assortment of other NGOs banded together to form an umbrella organization called the Framework Convention Alliance. The Alliance sponsored seminars for the delegates, lobbied them in the hallways, and put out an Alliance Bulletin every morning designed to sway delegates’ positions on various proposed treaty provisions. Much of the information distributed by the NGOs was valuable and accurate.’*

He went on to say, *‘the NGOs thus exerted tremendous influence over the course of the negotiations’* and *‘the NGOs worked the halls masterfully and, for all intents and purposes, filled the roles of deeply entrenched Washington insiders’.*

The contribution of civil society networks like the FCA are recognized in the FCTC Article 4.7 which states that the *‘participation of civil society is essential in achieving the objective of the Convention and its protocols’*.

## The FCA’s long-term impact

The FCA and its members helped increase the speed at which countries joined the FCTC, accelerating its entry into force. After the FCTC was adopted in 2003 and opened for signature by individual nations, the FCA began an advocacy campaign, including going door to door at permanent UN missions in New York City as the signing deadline approached in 2004. This resulted in numerous signatories of the FCTC. Later, to accelerate the entry into force of the FCTC, the FCA organized regional capacity building workshops in all six WHO regions, providing small grants to support ratification projects. The FCA’s activity at the national level was crucial to ensure that nations signed and ratified the treaty^3^. This may have contributed to how quickly the FCTC entered into force.

*‘I regard the Framework Convention on Tobacco Control as vitally important for global health and, without a doubt, the role of the FCA in motivating, organizing and coordinating the input of civil society into the treaty-making process was crucial to its success’,* said Gro Harlem Brundtland, Director-General of the World Health Organization (1998–2003) and former Prime Minister of Norway^4^.

## The FCA’s values: A model for civil society today

The way the FCA members were able to coalesce and work so effectively as a civil society community during the FCTC negotiations, with modest resources, remains a global example for civil society participation in UN treaty processes today. The FCA displayed an amazing ability to bring people together from all over the world and successfully influence the FCTC. The membership constructively debated positions internally and then reached consensus on which tobacco control measures to include in the treaty. Most importantly, members put individual and institutional egos aside to speak with one voice during the treaty negotiations^3^.

The FCA’s organizational process and structure is a model for civil society engagement in international treaty processes and has been covered in numerous academic articles^6^ and writings on effective coalitions.

As the ‘Advocacy for Impact: Lessons from Six Successful Campaigns’, a report commissioned by the Global Interdependence Initiative (a Program of the Aspen Institute)^7^, stated, *‘although the Alliance is funded by the West, the group has a decisively democratic and egalitarian feel where all strategies and tactics, and even funding decisions, are fiercely debated’.* This was a key priority for ASH as the Secretariat.

The 2005 report Advocacy for Impact described the FCA as *‘a vibrant network of more than 200 NGOs in 100 countries that are in continuous consultation and debate via a closed web link. Each member organization lobbies for the treaty and works on tobacco control in its own country, using strategies and tactics that are most effective in that situation’*^7^.

As the Advocacy for Impact report concluded when describing the FCA, *‘the global power of this loosely bound group derives from its ability to come together as a highly organized and unified force, as it did during the six rounds of international treaty negotiations. During these meetings, advocates from around the world met daily to coordinate their messages, events, and press releases for maximum impact. They used a wide range of advocacy tactics to influence the delegates to these meetings, including: providing solid research-based educational materials; publishing a daily newsletter of conference proceedings; organizing lunch meetings and performances; and orchestrating sensational, attention-getting displays like unveiling a “tobacco death clock”’*^7^.

The FCA was made up of doctors, lawyers, researchers, activists and public health experts that had put their lives on hold to challenge one of the most powerful industries in the world, backed by some of the most powerful governments in the world. ASH is proud to have been able to facilitate the unified participation of the FCA experts in civil society and beyond^3^.

## The FCTC today

Today, the FCTC has been joined by 182 countries and the European Union. Many have implemented its life saving measures. We even begin to see countries contemplating Article 2.1 of the FCTC, which recognizes that the FCTC Articles are the floor and not the ceiling of what is possible to achieve^3^.

In addition, we have the FCTC included as a ‘Target’ in the UN Sustainable Development Goals^8^, and in the mid-2010s, the FCTC began working in synergy with other UN Bodies around environmental, human rights, and development objectives. However, despite these successes and some progress at country level, the latest FCTC Implementation Report^9^ concluded that while results *‘show some positive developments… in recent years, as shown in previous global progress reports, the overall progress as measured by average implementation rates of the substantive Articles of the Convention was relatively slow’*.

In other words, countries are not maximizing the potential the FCTC offers.

## Looking forward

Today, thanks to our collective work, we have an evidence-based treaty joined by more than 180 countries that gives clear direction to the world on what to do to address the most dangerous consumer product on the market^3^. While we can rejoice in some victories for health, much more remains to be done given that the tobacco epidemic is still claiming millions of lives every year and damaging our environment^3^. The tobacco industry is the main obstacle to the implementation of the FCTC, and unfortunately, resources to implement and enforce the FCTC at country level are scarce, often due to a lack of whole of government engagement^3^. It is critical that we, as civil society, remain united and demand that governments work together, while excluding the tobacco industry, to accelerate comprehensive implementation of the FCTC and achieve its objective to *‘protect present and future generations from the devastating health, social, environmental and economic consequences of tobacco consumption and exposure to tobacco smoke’*^3^.

Yes, the tobacco industry is powerful, but countries are more powerful^3^.

No one should die because of tobacco, and the FCTC is a best practice tool to achieve not only health, but also environmental, human rights, and developmental objectives through a tobacco-free world vision. Let us learn from our progress as the FCA, and remain united to implement the FCTC and do just that^3^.

## Data Availability

Data sharing is not applicable to this article as no new data were created.
